# Engineering CRISPR/Cas9 for Multiplexed Recombinant Coagulation Factor Production

**DOI:** 10.3390/ijms23095090

**Published:** 2022-05-03

**Authors:** Colby J. Feser, Christopher J. Lees, Daniel T. Lammers, Megan J. Riddle, Jason R. Bingham, Matthew J. Eckert, Jakub Tolar, Mark J. Osborn

**Affiliations:** 1 Department of Pediatrics, Division of Blood and Marrow Transplantation, MMC 366 Mayo, 8366A, 420 Delaware Street SE, Minneapolis, MN 55455, USA; feser004@umn.edu (C.J.F.); leesx002@umn.edu (C.J.L.); baum0236@umn.edu (M.J.R.); tolar003@umn.edu (J.T.); 2 Department of General Surgery, Madigan Army Medical Center, 9040 Jackson Ave., Tacoma, WA 98431, USA; dtlammer@gmail.com (D.T.L.); jrpbingham@gmail.com (J.R.B.); matteckert1@gmail.com (M.J.E.); 3 Department of Surgery, University of North Carolina, 160 Dental Circle, Chapel Hill, NC 27599, USA

**Keywords:** CRISPR, recombinant protein, coagulation, multiplexing, fibrinogen

## Abstract

Current hemostatic agents are obtained from pooled plasma from multiple donors requiring costly pathogen screening and processing. Recombinant DNA-based production represents an engineering solution that could improve supply, uniformity, and safety. Current approaches are typically for single gene candidate peptides and often employ non-human cells. We devised an approach where multiple gene products could be produced from a single population of cells. We identified gene specific Synergistic Activation Mediators (SAM) from the CRISPR/Cas9 system for targeted overexpression of coagulation factors II, VII, IX, X, and fibrinogen. The components of the CRISPR-SAM system were expressed in Human Embryonic Kidney Cells (HEK293), and single (singleplex) or multi-gene (multiplex) upregulation was assessed by quantitative RT-PCR (qRT-PCR) and protein expression by ELISA analysis. Factor II, VII, IX, and X singleplex and multiplex activation resulted in 120–4700-fold and 60–680-fold increases in gene expression, respectively. Fibrinogen sub-unit gene activation resulted in a 1700–92,000-fold increases and 80–5500-fold increases in singleplex or multiplex approaches, respectively. ELISA analysis showed a concomitant upregulation of candidate gene products. Our findings demonstrate the capability of CRISPR/Cas9 SAMs for single or multi-agent production in human cells and represent an engineering advance that augments current recombinant peptide production techniques.

## 1. Introduction

Bleeding resulting from inherited disorders (e.g., hemophilia), trauma, surgery, and anti-coagulation therapy can require hemostatic coagulation products. Fresh frozen plasma (FFP), Cryoprecipitate (CRYO), Fibrinogen Concentrate (FC), and Prothrombin Complex Concentrate (PCC) are commonly employed agents to achieve/maintain hemostasis. Presently, plasma that is pooled from multiple, unrelated donors is the source of coagulative proteins, making them subject to supply chain shortages and costly pathogen screening. The lack of easily obtained, affordable, and safe sources of coagulation therapy products represent a significant gap in the field of resuscitative medicine.

Recombinant DNA (rDNA)-based production of hemostatic agents represents an engineering advance and is based on the introduction of a candidate gene into cells in vitro for supranormal gene expression and protein production. Mammalian cells are desirable for this process as most proteins are glycosylated in a species-specific manner. Accordingly, human proteins produced in non-human cells acquire glycosylation patterns that are differential than those deposited by human cells. The glycosylation profile of a protein is an important immune recognition mechanism for distinguishing self from non-self. As such, non-human produced proteins can be highly immunogenic and, in the case of resuscitative products that may be administered serially, can cause allosensitization [1].

**Figure 1:**
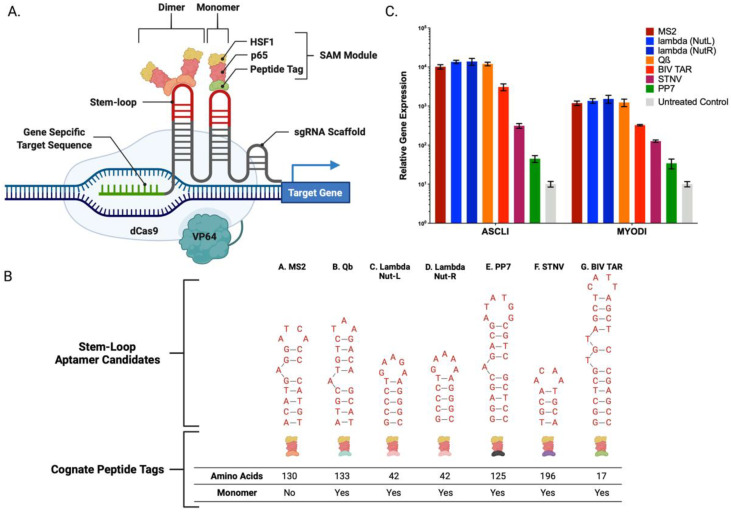
Synergistic Activation Mediator (SAM) Stem-Loop Aptamer and Cognate Recognition Peptide Candidates (**A**) Illustration of SAM system components. (**B**) Stem-loop aptamer and corresponding cognate recognition peptide candidates A. Bacteriophage MS2 (MS2) B. Bacteriophage Q-beta (Qb) C. Enterobacteria Phage Lambda Nut-L (Lambda Nut-L) D. Enterobacteria Phage Lambda Nut-R (Lambda Nut-R) E. Pseudomonas Phage PP7 (PP7) F. Satellite Tobacco Necrosis Virus (STNV) G. Bovine Immunodeficiency Virus TAR (BIV TAR). (**C**) mRNA-based gene expression analysis by qRT-PCR. Candidate aptamer and cognate peptide pairs were substituted into the MS2 SAM to initiate Ascl1 and MyoD1 gene overexpression in HEK293 cells (*n* = 3 individual experiments).

The Human Embryonic Kidney (HEK293) cell line has shown potential as an effective platform capable of producing recombinant coagulation factors VIII, IX, and fibrinogen [2,3]. By definition, recombinant technology employs DNA encoding candidate genes for introduction and production by cells. Often these genes are borne on plasmids, which are a small, circular species of DNA that can be isolated at high concentration and purity and, upon delivery to cells, facilitate supranormal gene expression of the candidate gene. Recently, Popovic et al. produced the three subunits of fibrinogen evaluating both a one and three plasmid approach to express recombinant fibrinogen protein [3]. Using this approach, they demonstrated overexpression of recombinant fibrinogen in HEK293 cells. Others have utilized similar plasmid-based approaches in non-human mammalian cell systems [4]. To extend these studies, we designed and optimized a transcriptome modulation approach employing the clustered regularly interspaced short palindromic repeats (CRISPR)/Cas9 system. CRISPR/Cas9 is a programmable DNA binding protein where Cas9 is directed to a user defined genomic target site using a small RNA transcript, termed a guide RNA (gRNA) [5]. gRNAs contain a ~15–20-bp sequence which corresponds to the target DNA sequence in the genome and binds via Watson–Crick base pairing. An ~80 nucleotide scaffold sequence is also present in the gRNA and is bound by Cas9 to form the functional complex [5]. Both the gRNA and Cas9 are amenable to modifications to enable enhanced properties such as genome, epigenome, and transcriptome editing [5,6]. We hypothesized that the programmability and engineering permissiveness of Cas9 would enable transcriptional upregulation of coagulation factor genes representing a novel approach for therapeutic peptide production. Our study design is based on generating gene targeting reagents from the CRISPR/Cas9 system to specifically upregulate the coagulation factor II, VII, IX, X, and fibrinogen genes. To achieve this, we employed a catalytically inactive Cas9 (dCas9) from Streptococcus pyogenes fused to the viral particle 64 (VP64) transcriptional activation domain that promotes gene transcription. The transcriptional potency of dCas9-VP64 can be potentiated with Synergistic activation modules (SAM) comprised of the Heat Shock Factor 1 (HSF1) and Nuclear Factor NF-kappa-B p65 subunit (p65) (Figure 1A) [6]. To accomplish this, scaffold sequences were incorporated into the gRNA to which RNA binding aptamers were incorporated [6]. An aptamer is a short single-stranded DNA or RNA species that can selectively bind a specific target such as a protein or peptide. We defined the optimal SAM sequences for single or multiplexed overexpression of PCC (factors II, VII, IX, and X) and fibrinogen in HEK293 cells. Our results represent a promising approach to produce a user defined recombinant peptide mixture in human cells. This cellular product represents a strategic reserve of therapeutic peptides with a reduced need for pathogen screening, as is required for pooled human plasma products.

## 2. Results

### 2.1. Clustered Regularly Interspaced Short Palindromic Repeats/Cas9-Synergistic Activation Module

We employed CRISPR/Cas9 SAMs positioned proximal to the transcriptional start sites of (factors II, VII, IX, and X) and fibrinogen for overexpression in HEK293 cells. The first described SAMs were derived from the bacteriophage MS2 and additional SAM aptamer, and cognate recognition peptide candidates were identified from similar bacteriophages [6]. We constructed and tested six additional p65:HSF-1 fused aptamers from: Bacteriophage Qb (QB), Pseudomonas Phage PP7 (PP7), Enterobacteria Phage Lambda (Lambda Nut-L and Nut-R), Satellite Tobacco Necrosis Virus (STNV), and Bovine Immunodeficiency Virus (BIV TAR) (Figure 1B,C and Appendix A Table A3) [7,8,9,10,11]. These optimization studies focused on the previously described Ascl1 and MyoD1 genes (Figure 1C) and we observed differential rates of activation for the unique aptamer candidates as assessed by quantitative RT-PCR (qRT-PCR) [6]. Of our candidates, the Lambda Nut-R aptamer/peptide, showed a 1.4-fold increase for Ascl1 and a 1.3-fold increase for MyoD1, respectively, compared to the previously reported MS2 system (Figure 1) [6]. Because Lambda Nut-R mediated the highest gene expression levels, it was therefore pursued as the lead candidate for the remainder of the study.

### 2.2. Lambda SAM Shows Potential for Prothrombin Complex Concentrate Production

**Figure 2:**
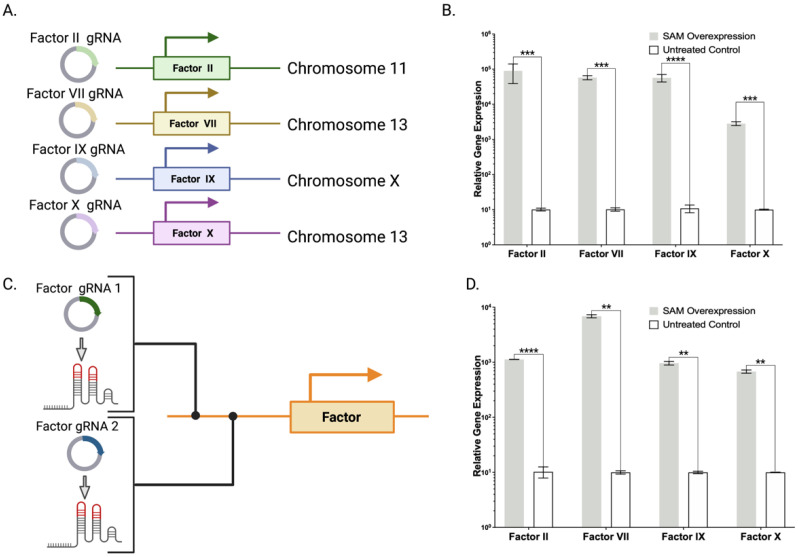
Lambda SAM singleplex and multiplex gene overexpression. (**A**) Factor II, VII, IX, and X gRNA design for singleplex activation. (**B**) mRNA-based gene expression analysis by qRT-PCR illustrating factor II, VII, IX and X transcript expression in Lambda SAM treated HEK293 cells compared to untreated WT cells (best gRNA pairs shown). (*n* = 3 independent experiments) (**C**) Paired gRNA overexpression approach. (**D**) mRNA-based gene expression analysis by qRT-PCR illustrating a simultaneous upregulation of factor II, VII, IX and X transcripts in Lambda SAM treated HEK293 cells compared to untreated WT cells (*n* = 2 independent experiments). *p* values were calculated using Student’s unpaired, two-sided *t* test to compare treated and untreated cells (** *p* ≤ 0.01, *** *p* ≤ 0.001, **** *p* ≤ 0.0001).

**Figure A1:**
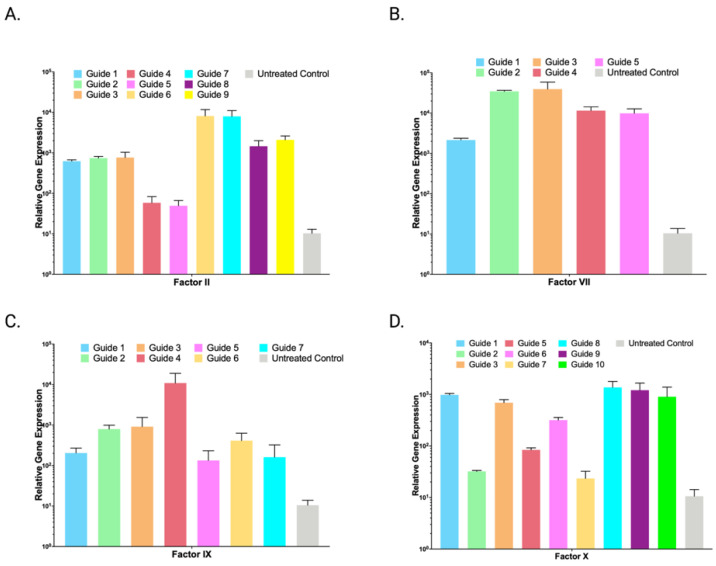
Lambda SAM singleplex single gRNA candidate evaluation of 4F-PCC. mRNA-based gene expression analysis by qRT-PCR illustrating factor (**A**). II, (**B**). VII, (**C**). IX, and (**D**). X transcript expression in Lambda SAM using single gRNA treated HEK293 cells compared to untreated WT cells. (*n* = 3 independent experiments).

**Figure A2:**
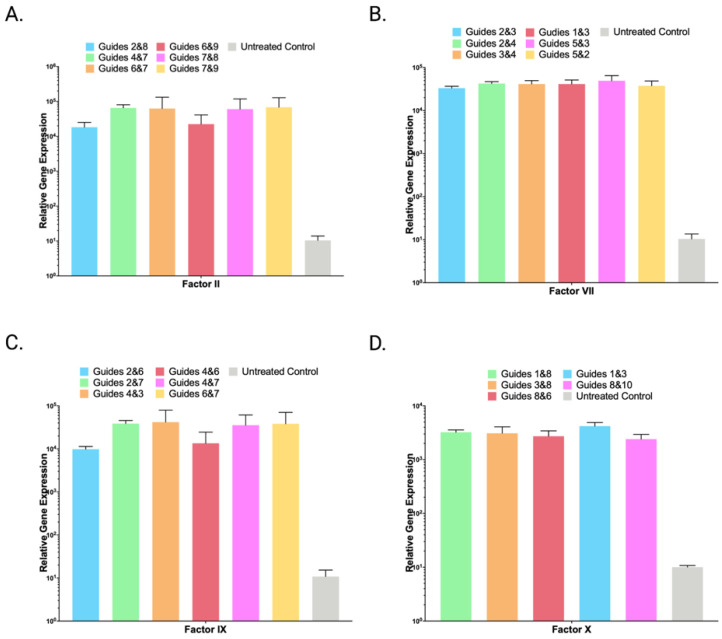
Lambda SAM singleplex paired gRNA candidate evaluation of 4F-PCC. mRNA-based gene expression analysis by qRT-PCR illustrating factor (**A**). II, (**B**). VII, (**C**). IX, and (**D**). X transcript expression in Lambda SAM using paired gRNA treated HEK293 cells compared to untreated WT cells. (*n* = 3 independent experiments).

Having optimized the components of the SAM system we next sought to define the positional effects of the Cas9-VP64 complex relative to the initiating methionine of coagulation factors II, VII, IX, and X that are key constituents of 4 factor-PCC (4F-PCC). We designed nine factor II, five factor VII, seven factor IX, and nine factor X candidate gRNAs, positioned within ~600 bp of the transcriptional start site (Figure 2A,B, and Appendix A Table A1). Each respective gene specific Lambda Nut-R gRNA was delivered into HEK293 cells along with Cas9-VP64, p65, and HSF1. Seventy-two hours after gene delivery, RNA was analyzed by qRT-PCR for quantification of the target transcript (Figure 2B and Appendix B Figure A1). From this approach we identified lead candidates and further optimized them for multiplex application by employing two guide RNAs (Figure 2C and Appendix B Figure A2). Two targeting gRNAs per gene improved activity over single gRNAs. The most active gRNA pairs displayed 280–8800-fold increases across each of the individual four coagulation factors compared to untreated HEK293 controls (Figure 2B and Appendix B Figure A2). Utilizing these optimized gRNA pairs under multiplexing conditions, we observed 60–680-fold increases in expression across the four PCC factors compared to untreated controls in HEK293 cells (Figure 2D).

### 2.3. Lambda SAM Mediated Protein Overexpression

**Figure 3:**
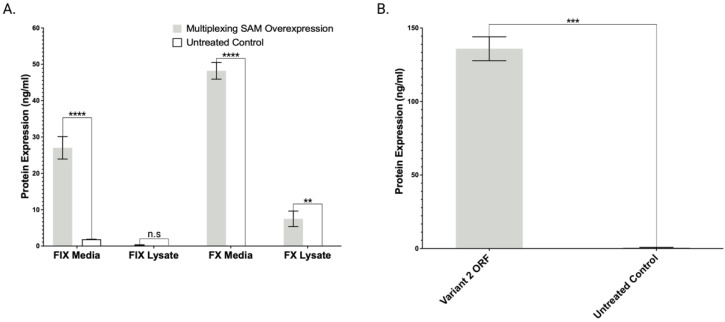
Lambda SAM overexpression of coagulation factors IX and X translates into secreted protein expression. (**A**) ELISA based protein quantification of transient SAM driven overexpression of coagulation factors IX and X or (**B**) Plasmid open reading frame driven overexpression of factor VII transcript variant 2 in HEK293 cells compared to untreated cells (*n* = 3 independent experiments. *p* values were calculated using Student’s unpaired, two-sided *t* test to compare treated and untreated cells (n.s. *p* > 0.05, ** *p* ≤ 0.01, *** *p* ≤ 0.001, **** *p* ≤ 0.0001).

**Figure 4:**
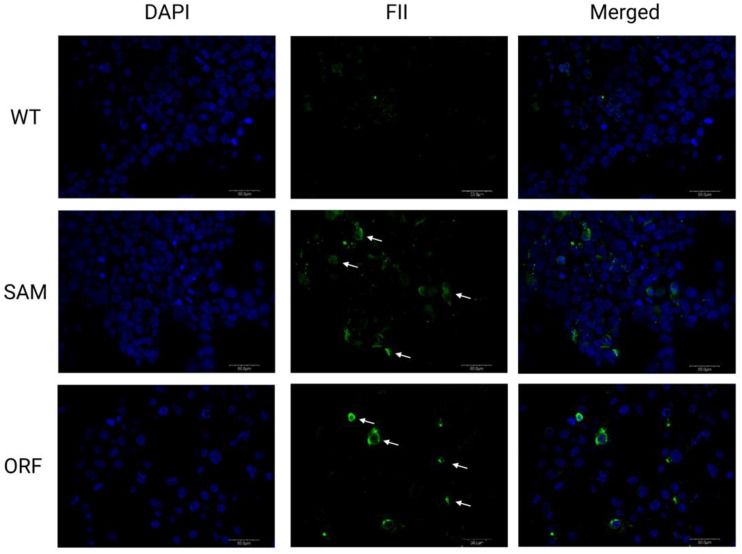
Immunofluorescent staining of factor II. Factor II expression is shown in untreated WT cells, SAM driven factor II overexpression, or overexpression from a plasmid-based open reading frame plasmid carrying the factor II sequence in HEK293 cells. Left panels represent DAPI counter stain (blue) followed by factor II stained with Alexa Fluor 488 (green) and the merged picture. Isotype controls (not pictured) were negative as expected.

Next, we undertook efforts to test whether the overexpression observed at the mRNA level translated into recombinant protein production. We transiently expressed the Lambda SAM using the optimized gRNA pairs for all four clotting factors using a singleplex approach with introduction of each pair into individual cell populations. Factors IX and X were detected in the supernatant of treated cells at levels of 27 ± 7 ng/mL (IX) and 48 ± 5 ng/mL (X) (Figure 3A). Interestingly factor II and factor VII, despite robust transcript detection, were not detected by ELISA (data not shown). To assess whether this was an assay or cell limitation, we employed plasmids expressing factor II and VII cDNAs in HEK293 cells. We observed robust production of factor VII, but not factor II, protein by ELISA (Figure 3B). We therefore performed immune fluorescence to show that SAM treatment resulted in factor II protein production (Figure 4). Given these data, we put forth an engineering model where in HEK293s we employed SAMs for factors II, IX, and X and a plasmid borne factor VII.

### 2.4. Lambda SAM Drives Activation of Multi-Domain Gene Fibrinogen

**Figure 5:**
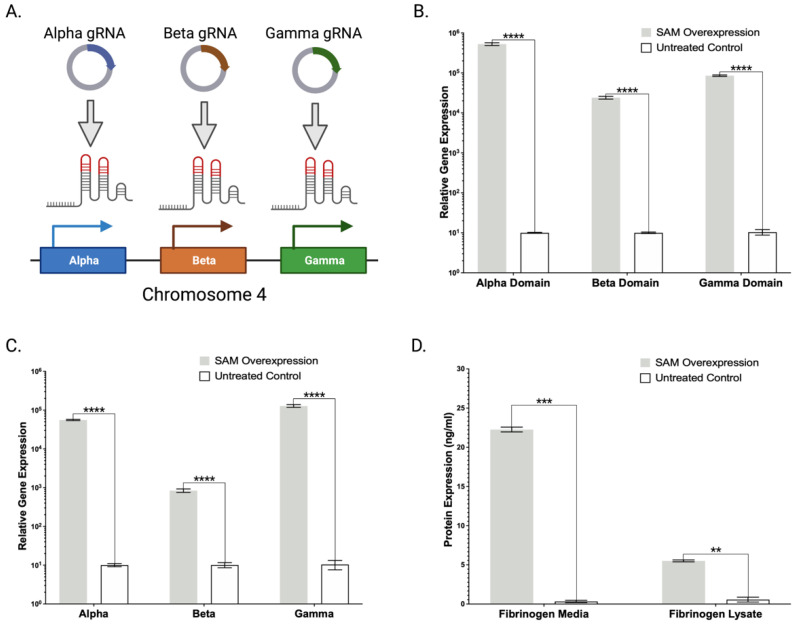
Lambda SAM singleplex and multiplex fibrinogen overexpression. (**A**) Fibrinogen Alpha, Beta, and Gamma chain gRNA design for gene activation. (**B**) mRNA-based gene expression analysis by qRT-PCR illustrating substantial increase in singleplex or (**C**) multiplex Alpha, Beta, and Gamma transcripts in Lambda SAM treated HEK293 cells compared to untreated WT cells. (*n* = 3 independent experiments) (**D**) ELISA analysis of SAM mediated multiplexing overexpression of Alpha, Beta, and Gamma domains resulting in fully assembled fibrinogen in HEK293 cells compared to untreated WT cells. *p* values were calculated using Student’s unpaired, two-sided *t* test to compare treated and untreated cells (** *p* ≤ 0.01, *** *p* ≤ 0.001, **** *p* ≤ 0.0001).

**Figure A3:**
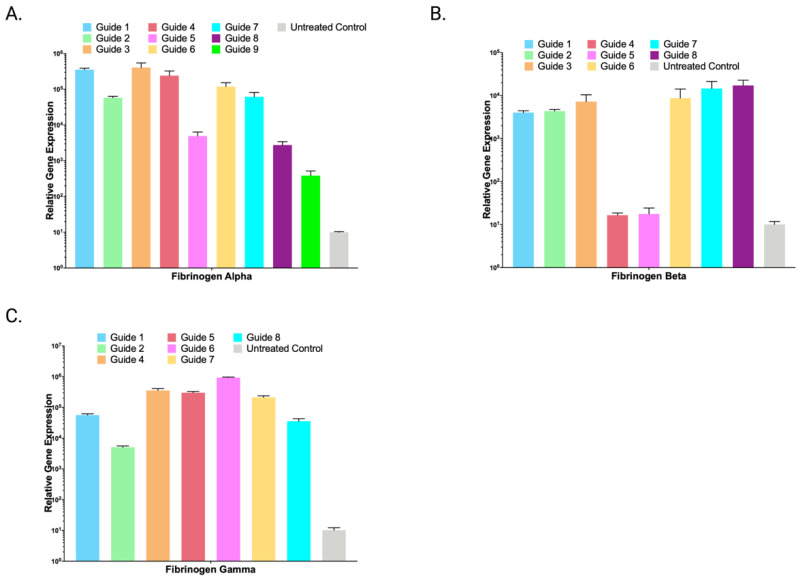
Lambda SAM singleplex single gRNA candidate evaluation of fibrinogen. mRNA-based gene expression analysis by qRT-PCR illustrating fibrinogen (**A**). Alpha, (**B**). Beta, and (**C**). Gamma transcript expression in Lambda SAM using single gRNA treated HEK293 cells compared to untreated WT cells. (*n* = 3 independent experiments).

Fibrinogen is an essential coagulation factor in the formation of stable clots, and commonly augments Factor II, VII, IX, and X PCC to achieve coagulation [12]. It is a complex protein made up of three distinct subunits (Alpha, Beta, and Gamma) which must be simultaneously expressed and subsequently assembled to yield functional fibrinogen. The multi gene/domain architecture of fibrinogen makes it ideally suited for multiplexed gene targeting using our strategy. We designed and evaluated nine Alpha, eight Beta, and seven Gamma gRNAs that delivered the Lambda SAM system into HEK293 cells (Figure 5A, Appendix A Table A1, Appendix B Figure A3). We observed a 1700–92,000-fold increase in expression across the three subunit genes with our best candidates (Figure 5B). We then applied the optimal gRNAs identified in this singleplex gene activation to multiplex targeting (Figure 5C). We observed an 80–5500-fold increase in expression across the three domains compared to the untreated control in HEK293 cells. Protein quantification studies showed fibrinogen predominantly as a secreted product with 22 ± 1 ng/mL present in the media (Figure 5D). These findings show that multi-domain genes such as fibrinogen can be multiplexed to drive simultaneous high level gene overexpression and protein overexpression.

## 3. Discussion

Recombinant DNA technology represents an engineering approach to support the in vitro production of resuscitative therapeutics. Human cells are desirable as they are capable of properly synthesizing and modifying complex human peptides making them desirable for recombinant methodologies. We chose HEK293 cells for their ease of culture, high gene transfer rates, and good manufacturing practice compatibility [13,14]. HEK293s have been applied for production of coagulation factors VIII, IX, and fibrinogen [2,3]. However, these efforts relied on the need for a one plasmid: one peptide engineering approach that can be cumbersome and costly as it requires a production procedure for each candidate plasmid.

Toward optimizing recombinant peptide production in human cells and streamlining the constituent components to coordinate supranormal peptide production, we sought to apply the CRISPR/Cas9 SAM system. A pioneering application of this system for gene upregulation has been the MS2 SAM CRISPR/Cas9 system [6]. We extended these studies to ascertain whether SAMs could be deployed as a novel engineering tool for resuscitative protein production. We first sought to optimize the SAM platform to maximize transcriptional activation noting that characteristics of the MS2 SAM architecture justified the undertaking. The MS2 aptamer recognition peptide is a comparatively large obligate dimer at a combined size of 260 amino acids [6]. This dimeric property represents the potential for steric effects in the context of the close proximity of stem loops incorporated into the gRNA. In addition, the monomeric:dimeric association/dissociation rate may be stochastic, leading to variable transcriptional activation. We defined the optimal SAM for overexpression by performing head-to-head comparisons of six significantly smaller peptide:aptamer pairs identified from other bacteriophages [7,8,9,10,11]. These comparison studies showed that the Lambda phage Nut-R candidate resulted in the highest gene expression levels for *AscII* and *MyoD1* (Figure 1). Interestingly, the remaining candidates showed a graded effect in terms of activation levels, representing an intriguing rheostatic potential for tuning gene expression to specified levels.

Having identified and streamlined the Lambda SAM architecture that mediated high level gene expression, we first employed them for the singleplex and multiplex gene activation of coagulation factors II, VII, IX, and X (Figure 2B,D). Factor IX and X showed robust mRNA and protein overexpression (Figure 3A). Because SAM mediated production of factors II and VII were recalcitrant to ELISA detection, we employed plasmid DNA for each open reading frame (ORF) to ascertain whether it was a methodological or biological hurdle. Factor VII but not factor II ORF plasmid enabled protein detection via ELISA. Therefore, to assess factor II production by SAMs we employed immunofluorescence and observed overexpression (Figure 4). Factor VII ORF plasmid resulted in demonstrable peptide upon analysis by ELISA (Figure 3B). We hypothesize that the FVII gene in the HEK293 genome may contain coding or transcript aberrations that enable FVII mRNA production but not protein. This is a key consideration for SAM application that, in our study design, we overcame by delivering a hybrid SAM:ORF plasmid mixture.

Toward fully designing, building, testing, and validating a SAM multiplex engineering scaffold, we expanded our studies to assess the production of fibrinogen. Coadministration of fibrinogen along with 4F-PCC is highly effective in the management of trauma-related bleeding, making it a desirable augmentative product for resuscitative medicine [15]. This complex protein made up of two sets of three polypeptide chains (Alpha, Beta, and Gamma) requires extensive disulfide bonds and glycosylation for proper assembly highlighting the importance of proper post-translational modifications and the need for capable human expression platforms. Transient plasmid-based approaches have been employed including a new study by Popovic et al. [3]. This study used a plasmid bearing all three fibrinogen cDNAs that transfected HEK293 and observed 8–12 ng/mL of fibrinogen. Building off this study, we designed and optimized SAM gRNAs to target the endogenous Alpha, Beta, and Gamma subunit genes of fibrinogen showing robust mRNA production (Figure 5B,C). We then showed by ELISA that SAM catalyzed gene expression facilitated 22 ± 1 ng/mL of secreted fibrinogen complex in HEK293 cells (Figure 5D). Our data complement the Popovic study and represent a novel and parallel engineering paradigm.

**Figure A4:**
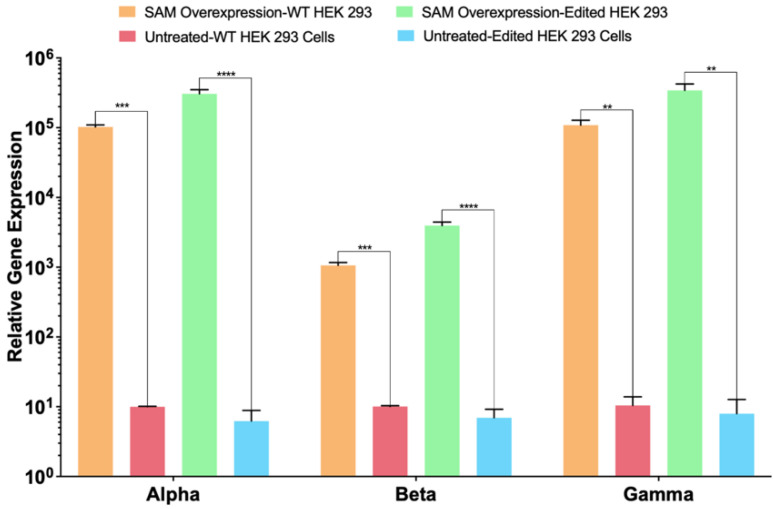
Edited dCas9-VP64/Lambda SAM HEK293 cell fibrinogen multiplex gene overexpression. mRNA-based gene expression analysis by qRT-PCR illustrating multiplexed fibrinogen Alpha, Beta, and Gamma transcripts in either Lambda SAM treated WT HEK293 cells or HEK293 cells stably expressing dCas9-VP64 and Lambda SAM compared to untreated WT cells. (*n* = 2 independent experiments). *p* values were calculated using Student’s unpaired, two-sided *t* test to compare treated and untreated cells (** *p* ≤ 0.01, *** *p* ≤ 0.001, **** *p* ≤ 0.0001).

Key considerations that are being pursued with our proof-of-concept data are purification and scaling. Popovic et al. showed that recombinant 293 produced fibrinogen could be isolated by traditional ethanol precipitation with enhanced purification by a highly selective novel ligand mimic binding motif [3]. This procedure could be assimilated into our workflow, and similar existent purification strategies employed for Factor II, VII, IX, and X and could likewise be leveraged for cellular purification. Toward promoting more efficient manufacturing and scaling, we generated dCas9 and Lambda SAM lentiviral vectors that were transduced into HEK293 cells. This cell line was then assessed by introducing the fibrinogen Alpha, Beta, and Gamma gRNA plasmids and compared with HEK293 cells, to which all component plasmids were delivered. Our results show that the edited cell line performed equivalently to the transient approach (Appendix B Figure A4). Together these results support development of an off-the-shelf cell population modular for production of diverse recombinant human therapeutic peptides.

In this study, we demonstrate proof-of-principle for CRISPR Cas9 based SAM mediated production of therapeutic gene products. Fibrinogen, factor II, VII, IX, and X prothrombin complex concentrate singleplex and multiplex supranormal gene expression in HEK293 cells was observed. These data represent an advance that adds to the engineering armamentarium that can be brought to bear to produce recombinant peptide(s) in human cells. In sum, the ability for SAMs to mediate supranormal expression of single or multiple genes simultaneously as we describe represents a facile system for cellular and protein engineering.

## 4. Materials and Methods

### 4.1. SAM Vector Assembly

Plasmids carrying dCas9-VP64 and MS2-p65-HSF1 were purchased from Addgene (Watertown, MA, USA). pMSCV-LTR-dCas9-VP64-BFP was a gift from Stanley Qi and Jonathan Weissman (Addgene plasmid #46912). Lenti MS2-P65-HSF1_Hygro was a gift from Feng Zhang (Addgene plasmid #61426). The gRNA scaffold plasmid was synthesized based on previous efforts [16]. The genes of interest were isolated and integrated into the mammalian expression vector pcDNA3.1 by Gibson Assembly using HiFi DNA Assembly Master Mix from NewEngland BioLabs (Ipswich, MA, USA) per the manufacturer’s instructions. Bacteriophage MS2 (MS2), Bacteriophage Q-beta (Qb), Pseudomonas Phage PP7 (PP7), Enterobacteria Phage Lambda (Lambda), Satellite Tobacco Necrosis Virus (STNV), and Bovine Immunodeficiency Virus Trans-Activation Response (BIV-TAR) stem loop and aptamer DNA sequences were synthesized by IDT (Coralville, IA, USA) and assembled into a gRNA backbone in a pcDNA3.1 expression in a similar fashion.

### 4.2. Cell Culture and Transfection Reagents

HEK293 cells, grown in DMEM complete media, were obtained from ATCC (CRL-1573) (Manassas, VA, USA). The adherent cells were grown as a monolayer in T75 cm^2^ Corning (Corning, NY, USA) cell culture treated flasks and detached with Trypsin-EDTA solution (0.25%) obtained from Invitrogen (Waltham, MA, USA). Transient transfections utilized Lipofectamine 2000 from Invitrogen seeding 100,000 cells per well in a Corning 24-well plate.

### 4.3. Quantitative RT-PCR

Total mRNA was isolated using the RNeasy Plus kit from Qiagen (Hilden, Germany). Equal amounts of RNA were reverse transcribed using Superscript VILO-IV from Invitrogen (Waltham, MA, USA). Gene expression analysis was carried out on a QuantStudio 3 real-time PCR system from Applied Biosystems (Waltham, MA, USA). Taqman gene expression master mix and probes (Appendix A Table A2) were used from Applied Biosystems. Expression data were normalized using GAPDH using the 2-ΔΔCT method.

### 4.4. Protein Quantification

Conditioned media and cell lysate were collected from treated HEK293 cells. Cells were lysed in Reporter Lysis Buffer from Promega (Madison, WI, USA) for 30 min before shearing through a 27.5-gauge needle. Both cell lysate and media were cleared of cellular debris by centrifugation before analysis. ELISA kits factor II (ab108909), VII (ab108829), factor IX (ab108831), factor X (ab108832), and fibrinogen (ab241383) were purchased from Abcam (Cambridge, UK). Analysis was conducted on a Tecan Infinite Microplate reader (Männedorf, Switzerland) using Magellan software.

### 4.5. Immunofluorescent Staining

WT HEK293 cells were seeded in a 24-well plate and transfected with plasmids containing either a factor II open reading frame or the elements of the SAM system (dCas9-VP64, Lambda-p65-HSF1, sgRNA) targeting factor II for overexpression. 48 h post-transfection cells were treated with trypsin, washed 1× with PBS, and seeded into gelatin-coated chamber slides from Corning (Corning, NY, USA). After 24 h cells were washed 3× with PBS and fixed in 4% paraformaldehyde followed by blocking with 3% BSA for 1 h. Cells were incubated at 4C overnight with a sheep anti-factor II antibody from Affinity Biologicals (SAFII-AP) (Ancaster, ON, Canada). Secondary Alexa Fluor 488 donkey anti-sheep antibody from Molecular Probes (A-11015) was incubated for 1 h at RT in the dark (Eugene, OR, USA). Slides were cover slipped with slowfade gold antifade DAPI, 4,6-diamidino-2-phenylindole (Invitrogen, Eugene, OR, USA) and examined by confocal fluorescence microscopy (Olympus BX61, Olympus Optical, Tokyo, Japan). Open reading frame plasmids for factor II (RC208589) and factor VII (RC213143) were obtained from Origene (Rockville, MD, USA).

### 4.6. Graphing, Statistical Analysis, and Images

All graphs were constructed using GraphPad Prism software v.8. Statistical analyses were performed using unpaired two-sided *t*-tests to compare the treated and untreated cells in transfection experiments. Figure schemas were created with BioRender.com accessed 10 April 2022.

## Data Availability

Additional data files are available upon request.

## Appendix A

**Table A1:** Complete list of unique gene specific 20 base pair gRNA targeting sequences.

Factor/Guide	Sequence	Proximity to Initiating Methionine (Base Pairs)
Factor II Guide 1	GGACAGGTCCTCCTGGGAGC	161
Factor II Guide 2	TCCTGTCCATCTCCACCATC	136
Factor II Guide 3	TCTAGGAGGAGTCACAAAAA	193
Factor II Guide 4	CGCTATATGTTAGGATTCGA	302
Factor II Guide 5	GGCGAGGGGCAACTTCCATC	533
Factor II Guide 6	CACACTATGGCGCACGTCCG	−14
Factor II Guide 7	TATCTACCCACCCGTCCCCA	112
Factor II Guide 8	AACATTAACCCAGAGGGGTC	40
Factor II Guide 9	ACATGAAGAAATCAGCGGAG	66
Factor VII Guide 1	ACCCCAGCTGTGCTGCAGAG	221
Factor VII Guide 2	GCTCAGTGGCTGGGCAGCAG	169
Factor VII Guide 3	TGCATGGCCACTGGCCGGCC	145
Factor VII Guide 4	AACTTTGCCCGTCAGTCCCA	44
Factor VII Guide 5	TTGCCAGCGTGCAGGTGTTA	253
Factor IX Guide 1	AAATTTCACCTCTGATTTCG	168
Factor IX Guide 2	CAGACTCAAATCAGCCACAG	201
Factor IX Guide 3	CTTGGGACAAGTGAAGAGAA	130
Factor IX Guide 4	CTCTCTGACAAAGATACGGT	304
Factor IX Guide 5	TCATCAGGATCTAGTGTTAG	607
Factor IX Guide 6	AATAGTCCAAAGACCCATTG	99
Factor IX Guide 7	CTTTCACAATCTGCTAGCAA	5
Factor X Guide 1	AGGCAGGGGAGTGACCACGC	138
Factor X Guide 2	GATGAGGATGAGGTTCTGCG	219
Factor X Guide 3	TCTGGCCGCCCAGCTCTGCC	192
Factor X Guide 4 ^1^	GTGGTCTCATGTCACGGGGG	527
Factor X Guide 5	AGGTGGTCTCATGTCACGGG	525
Factor X Guide 6	AGGCCGCTGGCAGTGAGCGT	419
Factor X Guide 7	ACAGTACTCGGCCACACCAT	−2
Factor X Guide 8	AGCAGGTCTCGGCTCCAATC	114
Factor X Guide 9	AGGTCTCGGCTCCAATCAGG	111
Factor X Guide 10	CGCTGGGAACACATCCACTC	155
Fibrinogen Alpha Guide 1	GGAGGAACAAAGGACGTAGA	142
Fibrinogen Alpha Guide 2	TCGGAAGCAAACAAGCTGAG	201
Fibrinogen Alpha Guide 3	TTCTGGGATACCAACAGCAT	162
Fibrinogen Alpha Guide 4	CAAGAGGTTTGGTTAATCAT	90
Fibrinogen Alpha Guide 5	TTTCAGCTGGAGTGCTCCTC	26
Fibrinogen Alpha Guide 6	CTTCCGATAAGCTGTTGCAA	214
Fibrinogen Alpha Guide 7	GAGATCTCCTAATTGTTGAC	254
Fibrinogen Alpha Guide 8	TGATTCTCCAGTCAACAATT	244
Fibrinogen Alpha Guide 9	GGAGGGTTGACTGTCTACAC	122
Fibrinogen Beta Guide 1	AAGATACACATCTCTCTTTG	264
Fibrinogen Beta Guide 2	GTAAATTTGACCTACTCACA	354
Fibrinogen Beta Guide 3	TTAGTGGTTGCCTTGTGAGT	367
Fibrinogen Beta Guide 4	TCTGAAGTCATTCCTAGCAG	46
Fibrinogen Beta Guide 5	ACAAGTAAATAAGCTTTGCT	149
Fibrinogen Beta Guide 6	CAGAGGACTCAGATATATAT	29
Fibrinogen Beta Guide 7	TCAGTTAAGTCTACATGAAA	−6
Fibrinogen Beta Guide 8	GGACAAAATGATGGGAAGTT	233
Fibrinogen Gamma Guide 1	AAGCATTATCCATTGCTGTG	292
Fibrinogen Gamma Guide 2	CTCCTTTTTGGCCCAGCTCA	231
Fibrinogen Gamma Guide 3 ^1^	TGATCCAAAATTATCTGCAA	263
Fibrinogen Gamma Guide 4	GGCCCCCAACCTATACTGTC	178
Fibrinogen Gamma Guide 5	AATGGCTGGAGCTGATCACG	103
Fibrinogen Gamma Guide 6	GGGAACCTGACAGTATAGGT	186
Fibrinogen Gamma Guide 7	ACGGGGCCTCCTTACCAGAA	120
Fibrinogen Gamma Guide 8	CACTACAAGGCTCGGAGCTC	16

^1^ Factor X Guide 4 and Fibrinogen Gamma Guide 3 were unable to be properly assembled despite repeated attempts likely due to formation of secondary structures inherent to the sequence.

**Table A2:** Complete list of qRT-PCR probes used for detection of transcripts of interest.

Gene	qRT-PCR Probe
Factor II	Hs01011988_m1
Factor VII	Hs01551992_m1
Factor IX	Hs01592597_m1
Factor X	Hs00984442_m1
GAPDH	Hs02758991_g1
Ascl1	Hs00269932_m1
MyoD1	Hs00159528_m1

**Table A3:** Complete list of SAM aptamer sequences and cognate peptides.

Candidate	Aptamer Sequence	Peptide Size(aa)
MS2	AACATGAGGATCACCCATGTC	130
Lambda Nut-L	GGGCCCTGAAGAAGGGCCC	42
Lambda Nut-R	GCCCTGAAAAAGGGC	42
Qb	AATGCATGTCTAAGACAGCAT	133
BIV TAR	GGCTCGTGTAGCTCATTAGCTCCGAGCC	17
STNV	CCTTTTCAAGACATGCAACAATGCACACAG	196
PP7	GGAGCAGACGATATGGCGTCGCTCC	125
